# N^6^-Methyladenosine RNA Demethylase FTO Promotes Gastric Cancer Metastasis by Down-Regulating the m6A Methylation of ITGB1

**DOI:** 10.3389/fonc.2021.681280

**Published:** 2021-07-01

**Authors:** Duo Wang, Xiujuan Qu, Wenqing Lu, Yizhe Wang, Yue Jin, Kezuo Hou, Bowen Yang, Ce Li, Jianfei Qi, Jiawen Xiao, Xiaofang Che, Yunpeng Liu

**Affiliations:** ^1^ Department of Medical Oncology, The First Hospital of China Medical University, Shenyang, China; ^2^ Key Laboratory of Anticancer Drugs and Biotherapy of Liaoning Province, The First Hospital of China Medical University, Shenyang, China; ^3^ Liaoning Province Clinical Research Center for Cancer, The First Hospital of China Medical University, Shenyang, China; ^4^ Key Laboratory of Precision Diagnosis and Treatment of Gastrointestinal Tumors, Ministry of Education, Shenyang, China; ^5^ Department of Respiratory and Infectious Disease of Geriatrics, The First Hospital of China Medical University, Shenyang, China; ^6^ Marlene and Stewart Greenebaum Comprehensive Cancer Center, University of Maryland, Baltimore, Baltimore, MD, United States; ^7^ Department of Medical Oncology, Shenyang Fifth People Hospital, Shenyang, China

**Keywords:** FTO, m6A, gastric cancer, metastasis, ITGB1

## Abstract

Abnormal RNA m6A methylation is known to lead to the occurrence and progression of multiple cancers including gastric cancer (GC). However, the integrative effects of all m6A methylation regulators on GC prognosis are unclear. Our research aimed to globally analyze the prognosis values of all 33 m6A RNA methylation regulators in GC by univariate and multivariate Cox regression analyses. Among all 33 m6A RNA methylation regulators, fat mass and obesity-associated protein (FTO), an m6A demethylase, was identified as a key prognostic risk factor on overall survival (OS) of GC patients. It was found that FTO could promote GC cell migration and invasion abilities, and we predicted that ITGB1 was a demethylated target of FTO. Knockdown (KD) of FTO significantly down-regulated ITGB1 expression at both mRNA and protein levels and augmented ITGB1 mRNA m6A modification level. Moreover, overexpression (OE) of ITGB1 could partially reverse FTO-KD-inhibited migration and invasion of GC cells. Our study found that FTO was an independent risk factor for overall survival (OS) of GC patients and FTO could promote GC metastasis by upregulating the expression of Integrin β1(ITGB1) *via* decreasing its m6A level. These results indicated that FTO can be a potent GC biomarker for prognosis prediction as well as a potential target in GC treatment.

## Introduction

Gastric cancer (GC) is the fifth most prevalent malignant cancer and the third leading cause of tumor-related mortality worldwide (1). Although significant progress has been made over the past few decades, overall survival (OS) of GC is still poor. In particular, the rate of five-year survival for patients diagnosed with distant-metastasis disease remains less than 5% (2). Therefore, it is vital to diagnose those patients with advanced GC as early as possible. Although a great deal of biomarkers have been developed to predict treatment efficacy, few could be finally used for clinical application. Therefore, it remains essential to identify more specific biomarkers to improve early diagnosis of GC.

For decades, epigenetic modifications including DNA methylation, histone modification and RNA methylation, have been widely reported to be related to the development of cancer. Recently, N6-methyladenosine (m6A), as the most prevalent RNA methylation modification, has attracted increasing attention (3–5). RNA m6A methylation, refers to a methyl group added to the N6 site of adenine, which regulated by methyltransferases (writers), demethylases (erasers), and m6A-binding proteins (readers) in a dynamic and reversible manner. The process of methylation is catalyzed by methyltransferases, including methyltransferase-like 3 (METTL3), METTL14 and their cofactors such as Wilms’ tumor 1-associated protein (WTAP) and RNA-binding motif protein 15 (RBM15); the removal of m6A methylation depends on erasers, including fat mass and obesity-associated protein (FTO) and AlkB homolog 5 (ALKBH5); and the recognition of m6A specific motif mediated by readers, such as YTH domain-containing proteins and IGF2BP1-3, is critical for the downstream functions. m6A methylation can regulate gene expression through mediating RNA degradation, splicing, translation, translocation and phase separation (6–15). Recently, accumulating studies have shown that aberrant m6A modification might be involved in carcinogenesis. For example, in gastric cancer, it has been reported that the methyltransferase METTL3 was essential for epithelial-mesenchymal transition (EMT) and metastasis, by enhancing ZMYM1 stability by increasing its m6A level to repress the transcription of E-cadherin (16); another methyltransferase KIAA1429 could enhance c‐Jun expression by stabilizing its mRNA in an m6A-dependent manner to promote cell proliferation (17). However, most of these studies are mainly focused on the role of a single methylation regulator. It is known that m6A methylation is a dynamic, reversible, and complicated process modulated by writers, erasers and readers. Therefore, evaluating the integrative role of all methylation regulators in cancers is more important than that of a single regulator. Although a bioinformatics study has established a 3-m6A-regulators-signature that can independently predict the prognosis of GC by analyzing 13 main m6A methylation modulators (18), the role of the other regulators in GC was not considered. Moreover, the lack of subsequent experimental work diminished the reliability of the analysis of the results. Therefore, it is still necessary to perform a global analysis of all m6A-methylation-regulators on the development of GC.

In this study, by analyzing the prognosis prediction values of 33 precisely reported m6A RNA methylation regulators in GC, we discovered that FTO was an independent risk factor for OS and FTO could promote GC metastasis by upregulating the expression of Integrin β1(ITGB1) by decreasing its m6A level. These results highlighted the significance of FTO in GC development, and demonstrated the potential of FTO as a novel biomarker in GC treatment.

## Materials and Methods

### Data Collection and Screening

RNA expression data of gastric cancer GC downloaded from The Cancer Genome Atlas (TCGA) database (http://cancergenome.nih.gov/) were used as the training dataset. Excluding the samples with incomplete information, the RNA expression data and clinical pathological parameters of 313 GC samples were obtained. After preprocessing the raw data for background correction and normalization, gene annotation was conducted by R package to get the gene expression information, and a total of 25330 genes were finally obtained. Gene expression data and corresponding clinical information of GSE62254 database obtained from the Gene Expression Omnibus (www.ncbi.nlm.nih.gov/geo/) were performed as an external validation. After the same pre-processing like in TCGA, totally 283 gastric cancer samples with complete information and 21642 genes were included in subsequent analysis.

### Construction of a Weighted Gene Co-Expression Network

Genes were ranked based on their standard deviation values from large to small, and a weight co-expression network (WGCNA) was accordingly constructed with the top 5,000 genes using the R package “WGCNA” (19). Scale independence and mean connectivity analysis of modules, which were essential for constructing network, were calculated by gradient test (power value ranging from 1 to 20). When the scale independence value was 0.9, the corresponding power value was selected. Next, a hierarchical clustering dendrogram of genes was generated using dissTOMGenes with similar expression modes were categorized into one module by the Dynamic Tree Cut function, with a criterion of the minimum size of 50 for the gene dendrogram. When the similarity of module epigengenes (MEs) was more than 0.75, modules were merged together.

### Identification of Key Modules Related to FTO Expression

The correlation coefficient between MEs and FTO expression levels were calculated to identify the relevant module. Gene significance (GS) was defined as the log10 transformation of the P-value (GS=lgP) between gene expression and the level of FTO expression in the linear regression. Moreover, module significance (MS) was described as the average GS of all the genes in one module. Consequently, the module with the highest absolute value of MS was defined as the key module significantly correlated to FTO expression.

### Functional Enrichment Analysis

To investigate the biological functions of genes in the selected key modules, Gene Ontology (GO) terms and Kyoto Encyclopedia of Genes and Genomes (KEGG) pathways were annotated and visualized using the R package “clusterprofiler” (20). GO terms involving biological processes (BP), cellular components (CC) and molecular functions (MF) and Kyoto Encyclopedia of Genes and Genomes (KEGG) pathways were picked up in accordance to the adjusted critical criterion *P*< 0.05. Furthermore, as a validation, gene set enrichment analysis (GSEA) was used to identify the biological function of FTO using a Java GSEA desktop application. Based on the median of FTO expression levels, all clinical samples were categorized into FTO-high-expression phenotype and FTO-low-expression phenotype to perform functional enrichment analysis. KEGG gene sets were performed as functional gene sets. The threshold values including *P*<0.05 and false discovery rate (FDR) <0.25 were used to calculate the statistical significance.

### Identification of Target Genes

The key target genes of FTO, which were identified in the pathways of both FA and ECM–receptor interaction, were screened by VENNY tool. Subsequently, with correlation analysis, the top five genes which were positively related to the expression of FTO were subjected to m6A methylation analysis by Whistle (https://whistle-epitranscriptome.com) and the m6Avar (http://m6avar.renlab.org). Later, the candidates which were predicted to have m6A modification sites both in Whistle and m6Avar, were analyzed using Kaplan-Meier survival analysis on OS of patients with gastric cancer.

### Survival Analysis

Univariate and multivariate analyses were evaluated by Cox proportional hazards regression models. The relationship between FTO expression and target genes expression was examined by Spearman correlation test. The OS was plotted by Kaplan-Meier survival analysis. All statistical analyses were calculated by R software (V 4.0.0) and GraphPad Prism 8. *P*<0.05 was regarded statistically significant.

### Cell Lines, Antibodies and Reagents

The gastric cancer cell lines BGC823, MGC803, HGC27 and AGS were purchased from the Chinese Academy of Sciences (Shanghai, China). MKN7 was from the Japanese Collection of Research Bioresources (JCRB Cell Bank, Osaka, Japan). SNU216 was obtained from the Korea Cell Line Bank (KCLB, Seoul, Korea). All cell lines except for AGS maintained in F-12K medium, were cultured in RPMI-1640 medium added with 10% heat-inactivated FBS. They were incubated at 37°C in saturated humidity with 5% CO2. Rabbit anti-FTO (#31687), anti-integrin β1 (#9699S), anti-GAPDH (#2118) and anti-LAMC1 (#4577) were obtained from Cell Signaling Technology (Danvers, MA, USA). Secondary goat anti-rabbit and goat anti-mouse antibodies were from Santa Cruz Biotechnology (Santa Cruz, CA, USA). Cycloleucine was purchased from Sigma (Sigma-Aldrich Co., St Louis, MO, USA). Matrigel was obtained from Corning (Corning Life Science, Tewksbury, MA, USA).

### Transfection

The specific siRNAs targeted to FTO and negative control siRNA (NC) were designed by JTS scientific (Wuhan, China). The coding strand of human FTO siRNA-1 was 5′- GUGGCAGUGUACAGUUAUATT-3′, and that of FTO siRNA-2 was 5′-GGCAAUCGAUACAGAAAGUTT-3′. A siRNA targeting sequence 5′-AATTCTCCGAACGTGTCACGT-3′ was used as a negative control. The plasmids ofpcDNA3.1-FLAG, pcDNA3.1-FTO and pcDNA3.1-ITGB1 were designed and constructed by Obio Technology Corp., Ltd (Shanghai, China). 0.1μM siRNAs, or 1mg/L plasmids were transfected into BGC823 and MGC803 cells (1.0 × 10^5^), using jetPRIME^®^ Transfection Reagent according to the manufacturer’s instructions.

### RNA Isolation and Quantitative Real-Time PCR

The extraction and reverse transcription of total RNA were conducted referred to the previous protocol (21). The relative RNA expression of FTO, ITGB1 and LAMC1 were calculated by the 2^−∆∆Ct^ method. 18S was used as the endogenous control. The primer sequences used were as follows:

FTO forward: 5′-AACACCAGGCTCTTTACGGTC-3′
FTO reverse: 5′-TGTCCGTTGTAGGATGAACCC-3′
ITGB1 forward: 5′-GCCGCGCGGAAAAGATGAA-3′;
ITGB1 reverse: 5′-TGCTGTTCCTTTGCTACGGT-3′;
LAMC1 forward: 5′-GCCGCGCGGAAAAGATGAA-3′;
LAMC1 reverse: 5′-TGCTGTTCCTTTGCTACGGT-3′;
18S forward: 5′-CCCGGGGAGGTAGTGACGAAAAAT-3′;
18S reverse: 5′-CGCCCGCCCGCTCCCAAGAT-3′.

### Methylated RNA Immunoprecipitation-qRT-PCR

Total RNAs (300μg) were extracted by TRIzol. The methylated RNA immunoprecipitation-qRT-PCR (MeRIP-qRT-PCR) assay was conducted with reference to the protocol of Magna MeRIP m6A Kit (Millipore, MA, USA, 17-10499). Enrichment of m6A containing mRNA was detected by qRT-PCR. The primer sequences were as same as those used in the previous qRT-PCR assay.

### Cell Viability Assay

MTT assay was used to evaluate cell viability. Firstly, 3×10^3^ cells/well transfected with NC siRNA and FTO siRNA were cultured in 96-well plates. After cell adherence, 20 μL MTT was added to each well at different time points (0h, 24h, 48h and 72h). Next, the cells were incubated for another 4h. The supernatant was then removed and 200 μL dimethylsulfoxide was added. Finally, the absorbance (A) at 570 nm was measured.

### Colony Formation Assay

500 cells/well were seeded into 12‐well plates. After 10‐14 days, colonies were fixed with 75% ethanol and then stained with crystal violet. Then, numbers of colonies in each well were counted.

### Western Blotting

The pretreated cells were dissolved in 1% Triton lysis buffer. After quantification, the protein samples were separated by SDS-polyacrylamide gel electrophoresis and then transferred onto PVDF membranes (Millipore, USA). After that, the PVDF membranes were blocked with 5% skimmed milk in TBS-T buffer, and then blotted with primary antibodies at 4°C overnight. Finally, after being hatched with secondary antibodies for 40 min, the membranes were exposed with enhanced chemiluminescence reagent and visualized using the Electrophoresis Gel Imaging Analysis System (DNR Bio-Imaging Systems, Israel).

### Transwell Assays

The transwell assays were conducted with 24-well chemotaxis plates (Corning, NY, United States). For migration assay, 2×10^4^ cells were placed into 200 μL serum-free 1640 medium onto the upper chambers and 500 μL1640 medium containing10% FBS were added to the bottom chambers. After incubation for 24 hours, the invaded cells were fixed in 75% ethanol, and then stained with Wright-Giemsa dye. For invasion assay, 50 μL matrigel at 1:30 dilution in serum-free 1640 medium were precoated onto the upper chamber before the cells were seeded. The following steps were the same as previously outlined in the migration assay.

### Statistical Analysis

Data are shown as means ± SD obtained from three independently repeated experiments. Student t-test was performed to calculate the expression differences between or among groups. *P*< 0.05 was regarded statistically significant.

## Results

### High Expression of FTO Predicted Poor Prognosis in Gastric Cancer

**Figure 1:**
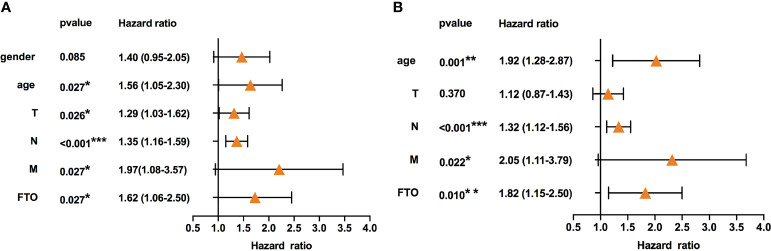
High expression of FTO indicated poor prognosis in gastric cancer. **(A)** Univariate Cox regression analyses of the association between clinicopathological factors (including FTO) and overall survival of patients in the TCGA datasets. **(B)** Multivariate Cox regression analyses of the association between clinicopathological factors (including FTO) and overall survival of patients in the TCGA datasets. *P < 0.05, **P < 0.01, and ***P < 0.001.

To screen out the prognostic risk factors of GC in m6A RNA methylation modulators, the effects of 33 m6A regulators on OS of GC were evaluated by univariate and multivariate Cox regression analysis using TCGA dataset. High expression of FTO and low expression of ALKBH5 and RBM15 were associated with poor OS by univariate Cox analysis (**Table 1**). FTO was then identified as an independent risk factor for poorer survival (HR=1.60, *P*=0.027, 95% CI: 1.10-2.50), whereas ALKBH5 was an independent protective factor for better survival (HR=0.61, *P*=0.026, 95% CI: 0.39-0.94) (**Table 2**). Next, to confirm the prognosis prediction value of FTO, FTO expression level and various clinicopathological parameters, were analyzed. The results of multivariate Cox regression analysis showed that the high expression of FTO (HR=1.82, P=0.010, 95% CI: 1.15-2.87), lymph node metastasis (HR=1.32, *P*<0.001, 95% CI: 1.12-1.56), distant metastasis (HR=2.05, *P*=0.022, 95% CI: 1.11-3.79) as well as older age (HR=1.92, *P*=0.001, 95% CI:1.28-2.87) were independent predictors (**Figures 1A, B**). Analysis of the GSE62254 dataset also verified the similar results (**Table S1**). Together, these analyses identified FTO as a prognostic risk factor of GC among all known RNA methylation regulators.

**Table 1:** The univariate Cox regression analysis of m6A RNA methylation regulators for OS in GC.

Gene	Coef	HR	95% CI	*P*	Gene	Coef	HR	95% CI	*P*
AGO2	-0.16	0.85	0.61-1.2	0.34	METTL16	-0.46	0.63	0.37-1.1	0.093
ALKBH5	-0.5	0.61	0.39-0.94	0.026	METTL3	-0.3	0.74	0.46-1.2	0.2
EIF3A	-0.099	0.91	0.61-1.3	0.62	METTL5	-0.09	0.91	0.63-1.3	0.63
EIF3B	0.11	1.1	0.8-1.6	0.53	RBM15	-0.5	0.61	0.4-0.93	0.021
EIF3C	0.18	1.2	0.79-1.8	0.39	RBM15B	-0.13	0.88	0.57-1.4	0.56
EIF3H	-0.098	0.91	0.62-1.3	0.61	RBMX	-0.066	0.94	0.59-1.5	0.78
ELAVL1	-0.23	0.79	0.48-1.3	0.37	TRMT112	-0.012	0.99	0.74-1.3	0.94
FMR1	-0.076	0.93	0.66-1.3	0.66	VIRMA	0.059	1.1	0.7-1.6	0.78
FTO	0.48	1.6	1.1-2.5	0.027	WTAP	-0.42	0.65	0.39-1.1	0.11
HNRNPA2B1	-0.3	0.74	0.5-1.1	0.13	YTHDC1	-0.31	0.74	0.41-1.3	0.31
HNRNPC	-0.22	0.8	0.5-1.3	0.36	YTHDC2	0.014	1	0.7-1.5	0.94
IGF2BP1	0.085	1.1	0.95-1.2	0.21	YTHDF1	-0.089	0.91	0.66-1.3	0.6
IGF2BP2	-0.014	0.99	0.83-1.2	0.87	YTHDF2	-0.45	0.64	0.38-1.1	0.089
IGF2BP3	-0.0061	0.99	0.82-1.2	0.95	YTHDF3	-0.033	0.97	0.65-1.4	0.87
LRPPRC	-0.085	0.92	0.67-1.3	0.6	ZC3H13	-0.047	0.95	0.71-1.3	0.75
MBNL1	0.12	1.1	0.83-1.6	0.44	ZCCHC4	-0.25	0.78	0.43-1.4	0.39
METTL14	-0.3	0.74	0.41-1.4	0.33					

**Table 2:** The univariate and multivariate Cox regression analysis of m6A RNA methylation regulators for OS in GC.

Gene	Univariate Cox				Multivariate Cox			
	Coef	HR	95% CI	*P*	Coef	HR	95% CI	*P*
ALKBH5	-0.5	0.61	0.39-0.94	0.026	-0.75	0.47	0.28-0.80	0.005
FTO	0.48	1.6	1.1-2.5	0.027	0.84	2.32	1.43-3.77	<0.001
RBM15	-0.5	0.61	0.4-0.93	0.021				

### FTO Promoted GC Cell Migration and Invasion

**Figure 2:**
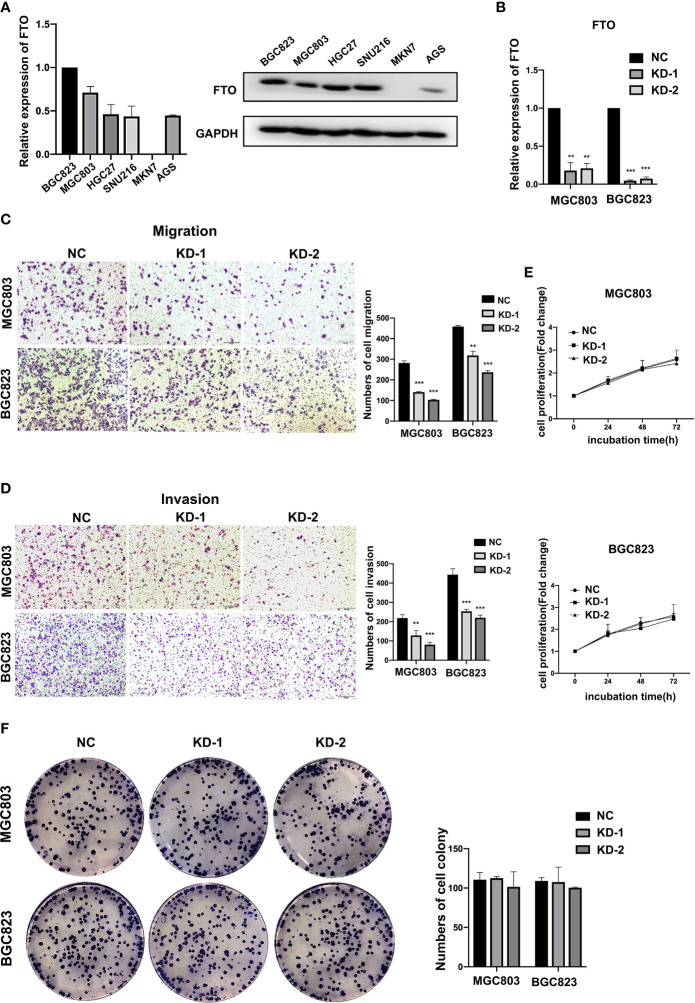
FTO promoted GC cell migration and invasion. **(A)** The relative mRNA and protein expression of FTO in different gastric cancer cell lines were detected by qRT-PCR and Western blot. **(B)** The knockdown efficiency of FTO in BGC823 and MGC803 cells was detected by qRT-PCR. **(C, D)** The migration and invasion ability of BGC823 and MGC803 cells after transfected with siNC or FTO siRNAs was examined by transwell assay (original magnification, 100×). The columns on the right are quantified by counting 3 fields, and presented as the mean ± standard deviation. **(E, F)** The proliferation ability of BGC823 and MGC803 cells after transfected with siNC or FTO siRNAs was examined by MTT and colony-formation assay. The columns are presented as the mean ± standard deviation. Data are presented as the mean ± SD of three independent experiments. **P < 0.01, ***P < 0.001. 18S was used as an internal control for all qRT-PCR experiments. GAPDH was used as an internal control for all western blot assays.

To determine whether FTO was involved in GC progression, GC cell lines were used to assess the effects of FTO on the ability of migration, invasion, and proliferation. After testing the expression of FTO among different cell lines at both RNA and protein levels (**Figure 2A**), BGC823 and MGC803 cells with a relatively high expression of FTO were transfected with two different siRNAs targeted to FTO (**Figure 2B**), and MKN7 cell with the lowest expression of FTO was transfected with FTO plasmid (**Figure S1A**). Knockdown of FTO (FTO-KD) in both GC cell lines significantly decreased migration and invasion (**Figures 2C, D**), but had no effect on proliferation (**Figures 2E, F**). On the contrary, overexpression of FTO (FTO-OE) enhanced the cell migration and invasion ability in MKN7 cell (**Figures S1B, C**). These results indicated that FTO acted as a key factor in migration and invasion of GC cells.

### Construction of Weight Co-Expression Modules With FTO Expression

**Figure 3:**
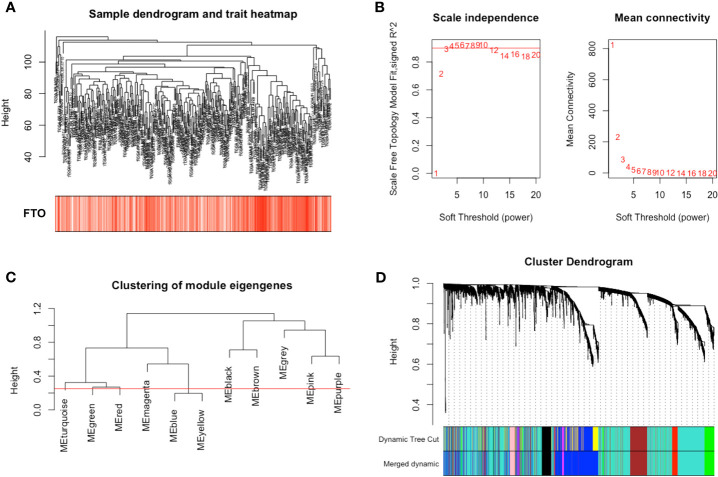
Construction of weight co-expression modules of gastric cancer with FTO expression. **(A)** The clustering was based on the expression data from TGCA. **(B)** The left figure showed the correlation of various soft-thresholding power and scale independence. The right figure showed the effect of soft threshold power values on mean connectivity. **(C)** Similar gene modules were merged according to the MEDissThres 0.25 (Red line) by calculating eigengenes of each module. **(D)** A hierarchical clustering dendrogram was conducted by the Dynamic Tree Cutting method. The first color band indicates the modules detected by dynamic tree cut. The second color band indicates the modules after merging similar modules.

To demonstrate the molecular mechanism of FTO on GC metastasis, we constructed a gene co-expression network using the R package “WGCNA”. Genes were subjected to cluster analysis using fashClust function (**Figure 3A**). At the approximate soft-thresholding power value 4, the minimum power for the scale-free topology fits index 0.9 (**Figure 3B**). Therefore, the power value 4 was chosen to build the scale-free network (**Figures S2A, B**). A hierarchical clustering tree was then generated using the Dynamic Tree Cutting method. According to the MEDissThres 0.25, similar modules were merged to finally result in 10 modules (**Figures 3C, D**).

### Functional Enrichment Analysis of FTO-Related Key Module

**Figure 4:**
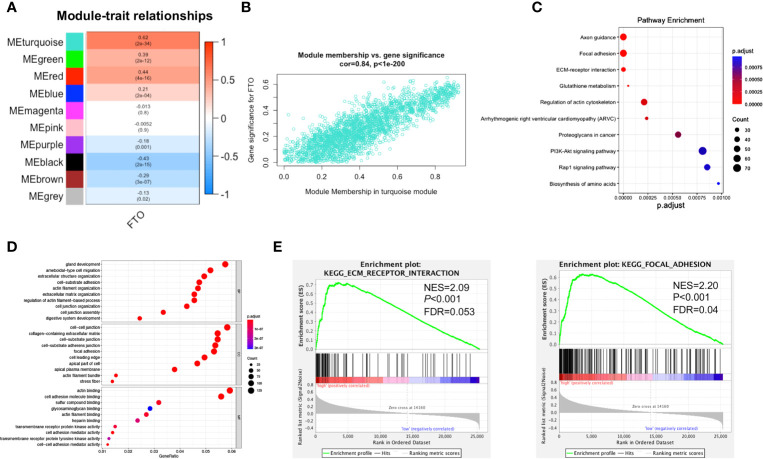
Functional enrichment analysis of FTO-related key module. **(A)** Heat map of the correlation between module eigengenes (ME) and the expression level of FTO. The turquoise module was the most positively correlated with FTO high expression. **(B)** Scatter plot of the correlation between genes in turquoise module and the expression level of FTO. **(C)** Significantly enriched KEGG pathways of turquoise module. **(D)** Significantly enriched GO annotations of turquoise module. **(E)** FTO high expression group enriched signaling pathways were analyzed using GSEA.

To investigate the key module related to FTO, the relationship between module eigengene and FTO expression level was analyzed. Among the ten modules, the turquoise module was the most positively associated with FTO expression (**Figures 4A, B**), indicating that the genes in turquoise module were closely related to FTO. Next, the genes in the turquoise module were further subject to enrichment analysis. The top 10 pathways uncovered multiple metastasis-related pathways, such as “FOCAL ADHESION (FA)” and “ECM-RECEPTOR INTERACTION” (**Table S2** and **Figure 4C**). Similarly, GO enrichment analysis also revealed that the genes in the turquoise module were closely associated with oncogenesis such as “cell-substrate adhesion”, “cell junction organization”, “cell-matrix adhesion” and “extracellular matrix organization” (**Figure 4D**). Furthermore, GSEA results also unveiled that high FTO expression was largely enriched in metastasis-related pathways, such as “FOCAL ADHESION (FA)” and “ECM-RECEPTOR INTERACTION” (**Figure 4E**). Finally, similar results of WGCNA were also obtained from the GSE62254 validation dataset that the genes in the black module, which was the most positively module associated with FTO expression, were closely related to metastasis-related pathways, such as “FOCAL ADHESION (FA)” and “ECM-RECEPTOR INTERACTION” (**Figures S3A–F**). All the above results suggested that high expression of FTO might promote GC metastasis by “FOCAL ADHESION (FA)” and “ECM-RECEPTOR INTERACTION” pathways.

### Screening of FTO Demethylated Target Genes Involved in GC Metastasis

**Figure 5:**
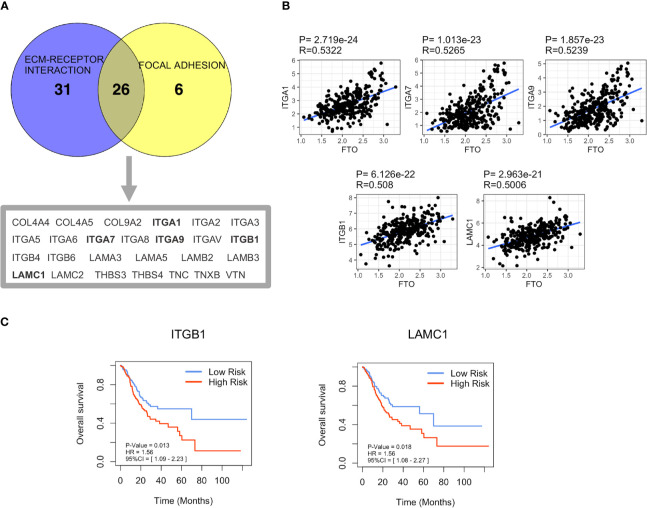
Screening of FTO demethylated target genes involved in GC metastasis. **(A)** The coexpression genes in “FOCAL ADHESION (FA)” and “ECM-RECEPTOR INTERACTION” pathways were screened by Venn diagram analysis. **(B)** The correlation between the expression of the top five coexpression genes and FTO was based on TCGA dataset. **(C)** Overall Survival (OS) of ITGB1 and LAMC1 in TCGA dataset was analyzed by Kaplan–Meier analysis.

It is known that m6A modification could down-regulate gene expression by promoting RNA degradation (8), and demethylase FTO may alleviate RNA degradation by decreasing the m6A modification level, thereby upregulating mRNA expression of target genes. Therefore, we hypothesized that FTO might upregulate the expression of genes related to “FOCAL ADHESION (FA)” and “ECM-RECEPTOR INTERACTION” pathways by decreasing its m6A modification level, thereby promoting GC metastasis. Hence, 26 genes overlapping in both “FOCAL ADHESION (FA)” and “ECM-RECEPTOR INTERACTION” pathways were screened as the candidates for FTO demethylated target genes using the VENNY tool (**Figure 5A**). After a correlation analysis between these 26 genes and FTO (**Table S3**), the top five genes, ITGA1 (R=0.5322, *P*<0.0001), ITGA7 (R=0.5265, *P*<0.0001), ITGA9 (R=0.5239, *P*<0.0001), ITGB1 (R=0.508, *P*<0.0001) and LAMC1 (R=0.5006, *P*<0.0001) (**Figure 5B**), were selected for further m6A methylation site prediction using Whistle (https://whistle-epitranscriptome.com/) and m6Avar (http://m6avar.renlab.org/index.html). The results showed that m6A modification sites were present only in ITGB1 and LAMC1, indicating ITGB1 and LAMCI might be the possible FTO demethylated target genes related to the promotion of metastasis (**Table 3**). Subsequently, Kaplan-Meier survival analysis indicated that patients in high expression of ITGB1 or LAMC1 group both presented shorter OS than that in the low expression group (**Figure 5C**). Analysis of the GSE62254 external dataset also obtained the similar results (**Figure S4**). Therefore, ITGB1 and LAMC1 were chosen as potential demethylated candidates by FTO for the further validation.

**Table 3:** m6A methylation sites prediction.

Gene	Whistle	m6AVar
ITGA1	None	Low
ITGA7	None	MeRIP-Seq (Medium)
ITGA9	None	Low
ITGB1	two	MeRIP-Seq (Medium)
LAMC1	seven	miCLIP (High)

### Identification of ITGB1 as an FTO Demethylated Target Gene in GC Cells

**Figure 6:**
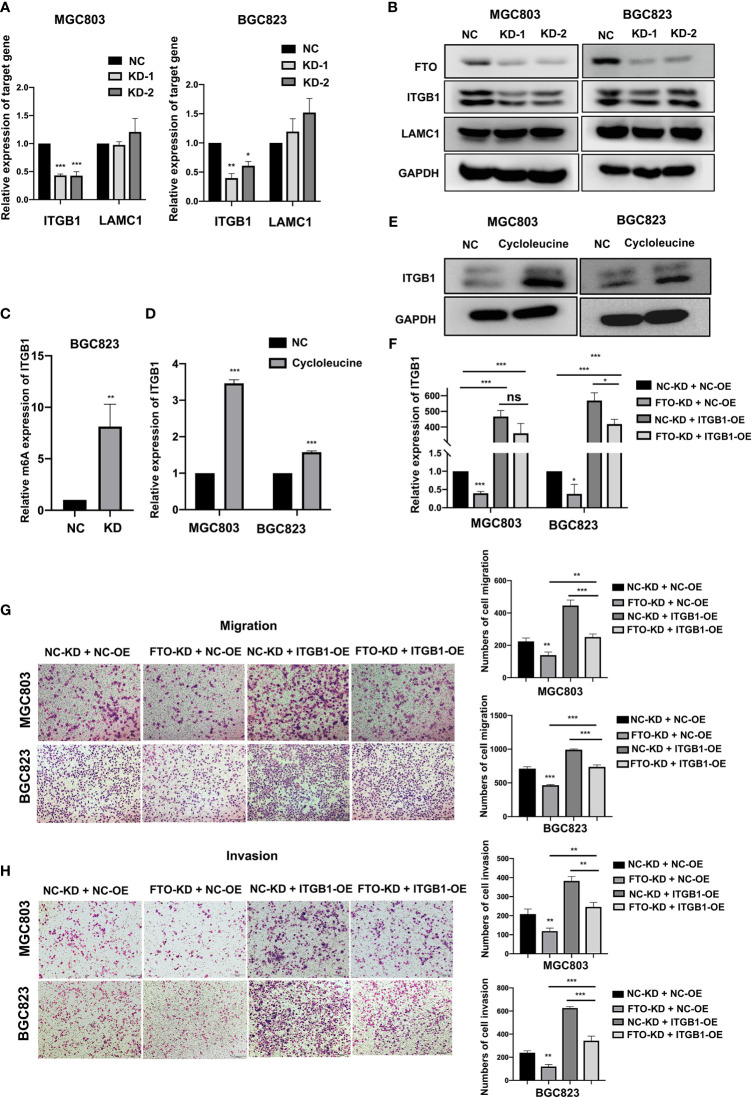
Identification of ITGB1 as FTO demethylated target gene in GC cells. **(A, B)** The relative mRNA and protein expression level of ITGB1 and LAMC1 in BGC823 and MGC803 cells after transfected with siNC or FTO siRNAs were detected by qRT-PCR and Western blot. **(C)** The m6A modification level of ITGB1 in BGC823 cell transfected with siNC or FTO siRNAs was detected by MeRIP-qRT-PCR. **(D, E)** The relative expression of ITGB1 in MGC803 and BGC823 cells after added with cycloleucine were detected by qRT-PCR and Western blot. **(F)** The relative expression of ITGB1 in BGC823 and MGC803 cells after co-transfected with siNC or FTO siRNAs and ITGB1 plasmids or empty vectors was detected by qRT-PCR. **(G, H)** The migration and invasion ability of BGC823 and MGC803 cells after co-transfected with siNC or FTO siRNAs and ITGB1 plasmids or empty vectors was examined by transwell assay (original magnification, 100×). The columns on the right are quantified by counting 3 fields, and presented as the mean ± standard deviation. Data are presented as the mean ± SD of three independent experiments. *P < 0.05, **P < 0.01, ***P < 0.001, ns, not statistically significant. 18S was used as an internal control for all qRT-PCR experiments. GAPDH was used as an internal control for all western blot assays.

To further confirm FTO demethylation target genes, the effect of FTO on the expression of ITGB1 and LAMC1 was assessed. FTO-KD dramatically attenuated the levels of ITGB1 mRNA and protein in MGC803 and BGC823 cells, but had no apparent effect on LAMC1 expression (**Figures 6A, B**), suggesting that ITGB1 might be the FTO demethylation target gene. The expression of ITGB1 was examined among different cell lines at both RNA and protein levels (**Figure S5**), showing consistency with that of FTO. Further MeRIP-qRT-PCR showed that FTO-KD significantly enhanced the m6A level of ITGB1 mRNA (**Figure 6C**), further supporting that ITGB1 was regulated by FTO-mediated m6A methylation. Subsequently, the expression of ITGB1 was examined in MGC803 and BGC823 cells after addition of cycloleucine, a small molecule inhibitor of m6A modification. The result indicated that ITGB1 was upregulated on both mRNA and protein level due to the lack of m6A methylation modification level (**Figures 6D, E**). Moreover, FTO-KD also decreased the phosphorylation level of focal adhesion kinase (FAK), the key downstream pathways of ITGB1 (**Figure S6**). To determine whether ITGB1 is a downstream effector of FTO in cell migration and invasion, ITGB1 was overexpressed in control and FTO-KD cells (**Figure 6F**). As a result, ITGB1-OE significantly attenuated the FTO-KD-suppressed migration and invasion (**Figures 6G, H**). Additionally, the phosphorylation level of focal adhesion kinase (FAK) decreased by FTO-KD was also partially reversed by ITGB1-OE (**Figure S7**). These data strongly indicated that FTO might promote GC cell migration and invasion *via* ITGB1-FAK pathway, by enhancing the ITGB1 expression in an m6A-dependent manner.

## Discussion

In this study, by systematically analyzing all 33 known m6A regulators, we identified that FTO acted as a key prognostic risk factor in GC. We further demonstrated that FTO could upregulate ITGB1 expression to promote GC metastasis by decreasing the m6A level of ITGB1 mRNA.

RNA m6A modification has been widely studied since the discovery of the first m6A demethylase (FTO) (22) and establishment of methylated RNA immunoprecipitation-sequencing techniques (23). m6A methylation is a complex biological process involving multiple methylation regulators. The same methylation regulator may result in different even controversial outcomes by different regulatory mechanisms in different cancers. Moreover, a bioinformatics analysis study across 33 cancer types demonstrated that all of the m6A regulators were tightly relevant to the OS of patients in at least one cancer type and to the alterations of cancer pathways. Furthermore, the distribution of m6A modifications varied with the types of cancers; the prognostic role of m6A modulators may even be inconsistent among subtypes in one type of cancer (24). Therefore, analyzing the integrative roles of all methylation regulators in various cancers may be more advantageous than analyzing one or a few regulators. To date, few studies have comprehensively explored the effects of m6A regulators. Su et al. established a 3-m6A-methylation-regulators-signature to predict the prognosis of GC after analyzing13 m6A regulators (18); similarly, the expression of seven main regulators were merged as W, R and E signatures to represent the m6A modification level (25). However, the roles of the other m6A modulators in gastric cancer were not examined in those previous studies. Therefore, in our study, we investigated the prognostic roles of all 33 precisely reported m6A RNA methylation modulators in two datasets of GC (TCGA, GSE62254). FTO was identified as the most potent prognostic risk factor among the 33 regulators by univariate and multivariate Cox regression analysis. High expression of FTO was correlated to poorer prognosis in GC. Our result is consistent with a previous study (18), and further demonstrated the dominant role of FTO in affecting the prognosis of GC among 33 regulators.

Indeed, the roles of methyltransferases or demethylases in cancer are not always invariable. Accumulating studies recently have reported that FTO acts as an oncogene in various cancers such as glioblastoma, leukemia, melanoma, and lung cancer (26–35). In glioblastoma stem cells, inhibition of FTO suppressed stem cell growth, self-renewal as well as tumor progression (26). Elsewhere, research into melanoma also unveiled that FTO increased cell proliferation and migration; FTO knockdown decreases the expression of melanoma-related genes *via* an m6A-YTHDF2-mediated-manner (28). However, several other researchers have drawn contradictory conclusions to the effect that FTO played a tumor-suppressing role in ccRCC and ovarian cancer (36, 37). A similar inconsistency was also found in METTL3 and METTL14. They have been also controversially reported to act as oncogenic and tumor-suppressive genes in glioblastoma and hepatocellular carcinoma (26, 38–40). In this study, we found that FTO-KD effectively suppressed the migration and invasion abilities of GC cells, but could not affect the proliferation of both BGC823 and MGC803 cells, and FTO-OE enhanced the cell migration and invasion ability in MKN7 cells, which were partially consistent with some previous researches (25, 41, 42), suggesting that FTO might lead to poor prognosis by promoting metastasis in GC. As methylation modification was widely distributed in thousands of genes, the same m6A modulators-mediated different target genes m6A modification might be one of the key reasons that they play different roles in different cancers.

We identified ITGB1 and LAMC1 as possible demethylated targets of FTO by using WGCNA and GSEA analyses, and m6A modification site prediction. Subsequently, knockdown of FTO dramatically abolished ITGB1 mRNA and protein expression, but not LAMC1. Although ITGB1 and LAMC1 were both tightly related to the prognosis of gastric cancer, it was suggested that LAMC1 might be involved in a more complex molecular mechanism, rather than being directly regulated by FTO. Furthermore, MeRIP-qRT-PCR revealed that silencing of FTO significantly enhanced the m6A level of ITGB1 mRNA, and the expression of ITGB1 was upregulated on both mRNA and protein level after the treatment with cycloleucine, a small molecule inhibitor of m6A modification, ultimately supporting the concept whereby ITGB1 is modulated by FTO-mediated m6A methylation. ITGB1 binding to α subunits to form 12 integrin receptors, interacts with multiple extracellular matrix molecules, which could activate the formation of focal adhesion complexes and play a vital role in the promotion and invasion of cancer (43–45). Therefore, we examined the possible downstream pathway of ITGB1 in GC cells, and found that the level of p-FAK was decreased by FTO-KD and could be further partially reversed by ITGB1-OE, indicating that FTO might promote GC cell migration and invasion *via* ITGB1-FAK pathway. ITGB1-OE has been extensively reported to be able to promote tumor metastasis and progression in various types of cancers (46–48). To date, the m6A modification mechanisms of ITGB1 have not been widely studied. A recent study reported that METTL3 could stabilize ITGB1 mRNA *via* an m6A-HuR-dependent mechanism to promote bone metastasis in prostate cancer (49). Our result suggested that ITGB1 could be epigenetically regulated by m6A modification mediated by FTO. The m6A modification site of ITGB1 mRNA remains unknown. It is reported that m6A modification sites are generally located in the 3′-UTR of mRNA. In future work, MeRIP-sequencing is needed to determine whether the m6A modification site is distributed in the 3′-UTR or other parts of ITGB1 mRNA. Moreover, FTO may potentially regulate the many mRNA targets to promote GC metastasis. Therefore, ITGB1 should not be the only target modulated by FTO-demethylation. Future research is needed to further identify other FTO targets underlying GC metastasis.

In summary, we found that FTO was the most potent prognostic risk factor among all the 33 m6A regulators, and demonstrated that FTO could promote GC metastasis by augmenting ITGB1 expression likely *via* m6A modification. Our findings supported that FTO may act as a potential GC biomarker and shed light on future cancer.

## Data Availability Statement

The datasets presented in this study can be found in online repositories. The names of the repository/repositories and accession number(s) can be found in the article/**Supplementary Material**.

## Author Contributions

YL and XC designed the study and revised the manuscript. DW did the majority of experiments and drafted the manuscript. WL and BY preprocessed and interpreted the data. YJ performed partial qRT-PCR experiment. XQ, CL, YW, JQ, and JX conducted the experimental guidance. All authors contributed to the article and approved the submitted version.

## Funding

This study was supported by the National Key Research and Development Program of China (2017YFC1308900), National Natural Science Foundation of China (No. 81972751,81972331), Technological Special Project of Liaoning Province of China (2019020176-JH1/103), The Key Research and Development Program of Liaoning Province (2018225060), and Science and Technology Plan Project of Shenyang City (19-112-4-099).

## Conflict of Interest

The authors declare that the research was conducted in the absence of any commercial or financial relationships that could be construed as a potential conflict of interest.

## Abbreviations

GC, gastric cancer; OS, overall survival; ALKBH5, AlkB Homolog 5; m6A, N6-methyladenosine; FTO, obesity-associated protein; METTL3, methyltransferase-like 3; METTL5, methyltransferase like 5; METTL14, methyltransferase like 14; METTL16, methyltransferase like 16; WTAP, Wilms’ tumor 1-associated protein; VIRMA, Vir like m6A methyltransferase associated; RBM15, RNA-binding motif protein 15; RBM15B, RNA binding motif protein 15B; ZC3H13, zinc finger CCCH-type containing 13; YTHDF1, YTH N(6)-methyladenosine RNA binding protein 1; YTHDF2, YTH N(6)-methyladenosine RNA binding protein 2; YTHDF3, YTH N(6)-methyladenosine RNA binding protein 3; YTHDC1, YTH domain containing 1; YTHDC2, YTH domain containing 2; AGO2, argonaute RISC catalytic component 2; RBMX, RNA binding motif protein, X-linked; ELAVL1, ELAV like RNA binding protein 1; HNRNPA2B1, heterogeneous nuclear ribonucleoprotein A2/B1; HNRNPC, heterogeneous nuclear ribonucleoprotein C (C1/C2), FMR1, fragile X mental retardation 1; LRPPRC, leucine-rich pentatricopeptide repeat containing; IGF2BP1, insulin-like growth factor 2 mRNA binding protein 1; IGF2BP2, insulin-like growth factor 2 mRNA binding protein 2; IGF2BP3, insulin-like growth factor 2 mRNA binding protein 3; EIF3A, eukaryotic translation initiation factor 3, subunit A; EIF3B, eukaryotic translation initiation factor 3, subunit B; EIF3C, eukaryotic translation initiation factor 3, subunit C; EIF3H, eukaryotic translation initiation factor 3, subunit H; ZCCHC4, zinc finger, CCHC domain containing 4; TRMT112, tRNA methyltransferase 11-2 homolog; MBNL1 Muscleblind-like protein 1;ITGB1, Integrin β1; TCGA, the Cancer Genome Atlas; WGCNA, weight co-expression network; KEGG, Kyoto Encyclopedia of Genes and Genomes; GO, Gene Ontology; GSEA, gene set enrichment analysis; KD, knockdown; OE, overexpression; NC, negative control; qRT-PCR, quantitative real-time; PVDF, polyvinylidene difluoride.
