# Supplementary material for: N6-Methyladenosine RNA Demethylase FTO Promotes Gastric Cancer Metastasis by Down-Regulating the m6A Methylation of ITGB1
Source: Front Oncol. 2021 Jul 1;11:681280. doi: 10.3389/fonc.2021.681280 (PMC8282183; doi:10.3389/fonc.2021.681280)
Supplement: Supplementary file 1 [file DataSheet_1.pdf]

Supplementary Material

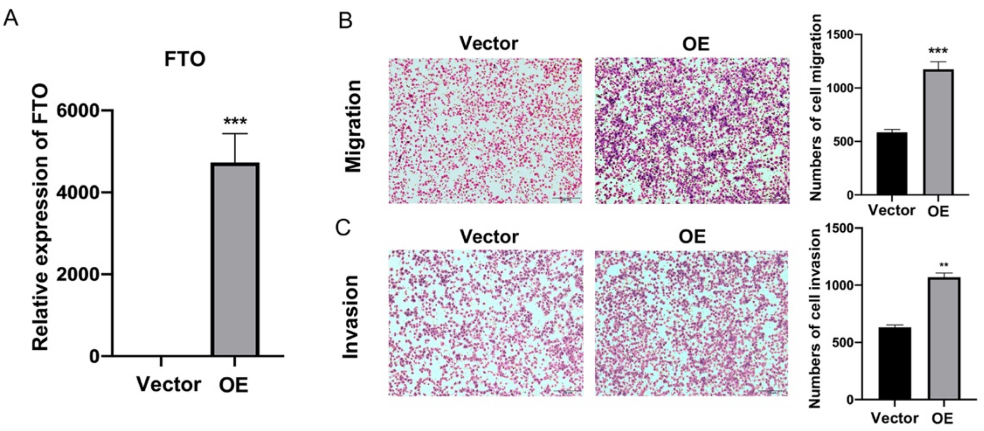

Figure S1 Overexpression of FTO promoted GC cell migration and invasion. **(A)** The overexpression efficiency of FTO in MKN7 cell was detected by qRT-PCR. **(B-C)** The migration and invasion ability of MKN7 cell after transfected with FTO overexpression plasmid was examined by transwell assay (original magnification, 100×). The columns on the right are quantified by counting 3 fields, and presented as the mean ± standard deviation. *P<0.05, **P<0.01.

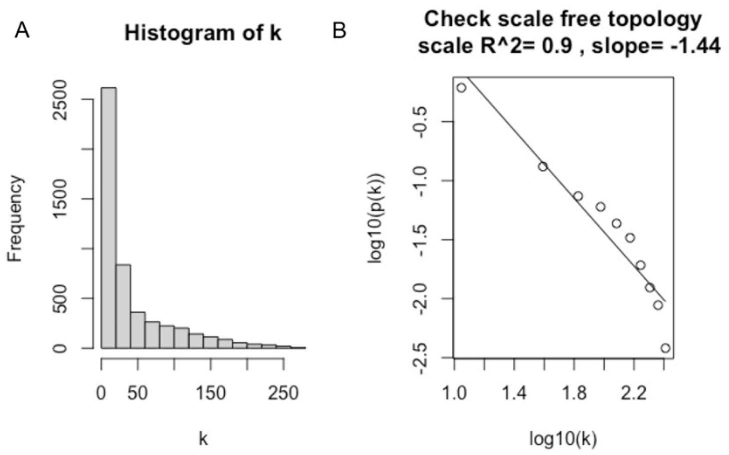

Figure S2 Determination of soft-thresholding power in the weighted gene co-expression network analysis (WGCNA). **(A)** The histogram of connectivity distribution when $\beta = 4$. **(B)** The check scale free topology when $\beta = 4$.

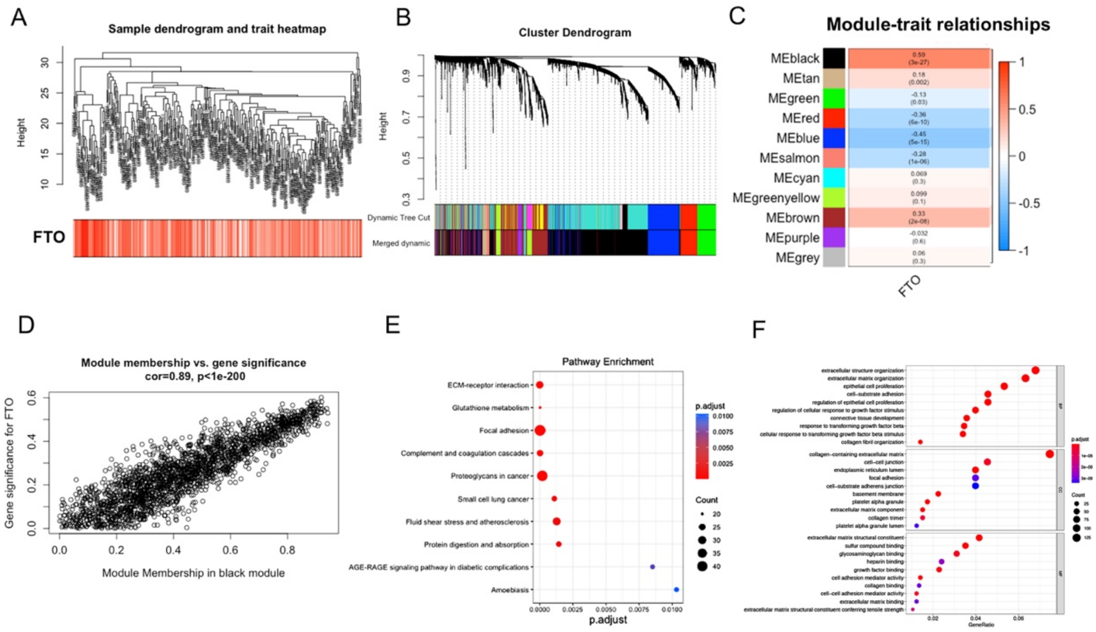

Figure S3 Construction of weight co-expression modules with FTO expression and functional enrichment analysis of FTO-related key module in GSE62254 dataset. **(A)** The clustering was based on the expression data from GSE62254 dataset. **(B)** A hierarchical clustering dendrogram was conducted by the Dynamic Tree Cutting method. The first color band indicates the modules detected by dynamic tree cut. The second color band indicates the modules after merging similar modules. **(C)** Heat map of the correlation between module eigengenes (ME) and the expression level of FTO. The black module was the most positively correlated with FTO high expression. **(D)** Scatter plot of the correlation between genes in black module and the expression level of FTO. **(E)** Significantly enriched KEGG pathways of black module. **(F)** Significantly enriched GO annotations of black module.

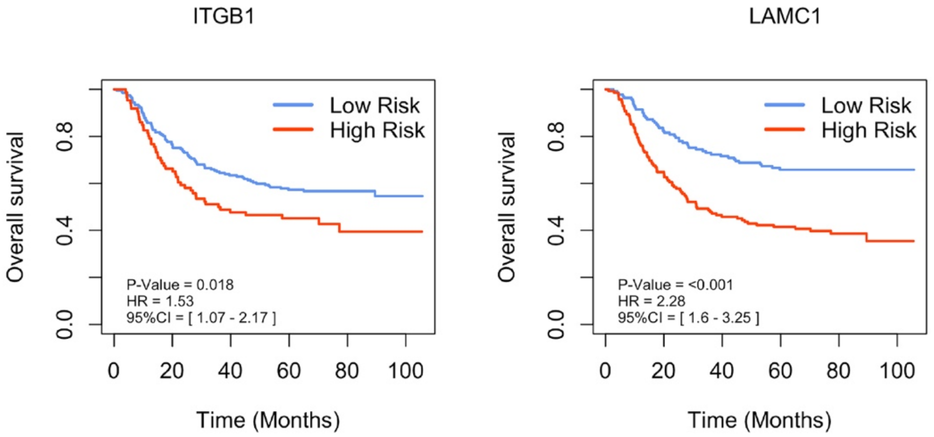

Figure S4 Overall Survival (OS) of ITGB1 and LAMC1 in GSE62254 dataset was analyzed by Kaplan–Meier analysis.

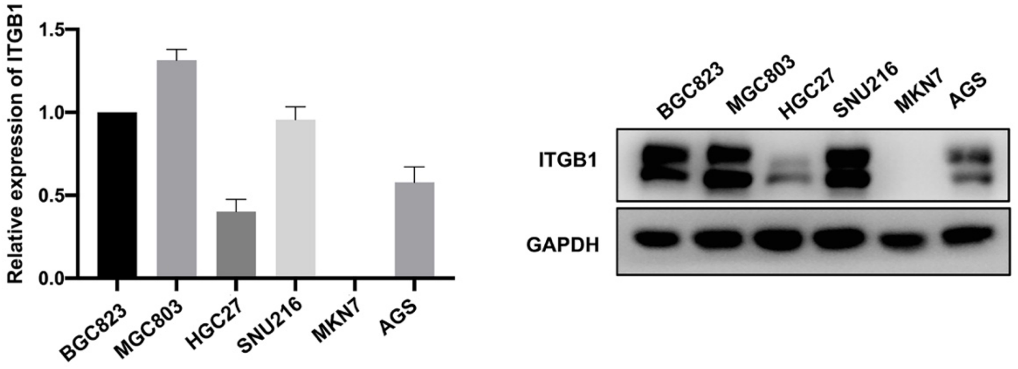

Figure S5 The relative mRNA and protein expression of ITGB1 in different gastric cancer cell lines were detected by qRT-PCR and Western blot. Data are presented as the mean ± SD of three independent experiments. **P*<0.05, ***P*<0.01.18S was used as an internal control for all qRT-PCR experiments. GAPDH was used as an internal control for all western blot assays.

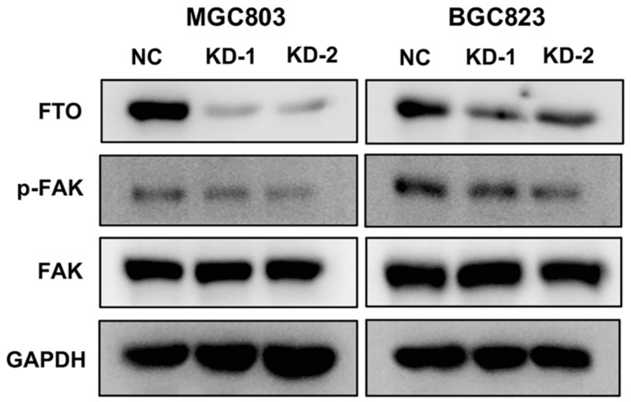

Figure S6 The downstream pathway protein in GC cells after transfected with siNC or FTO siRNAs were detected by Western blot. GAPDH was used as an internal control for all western blot assays.

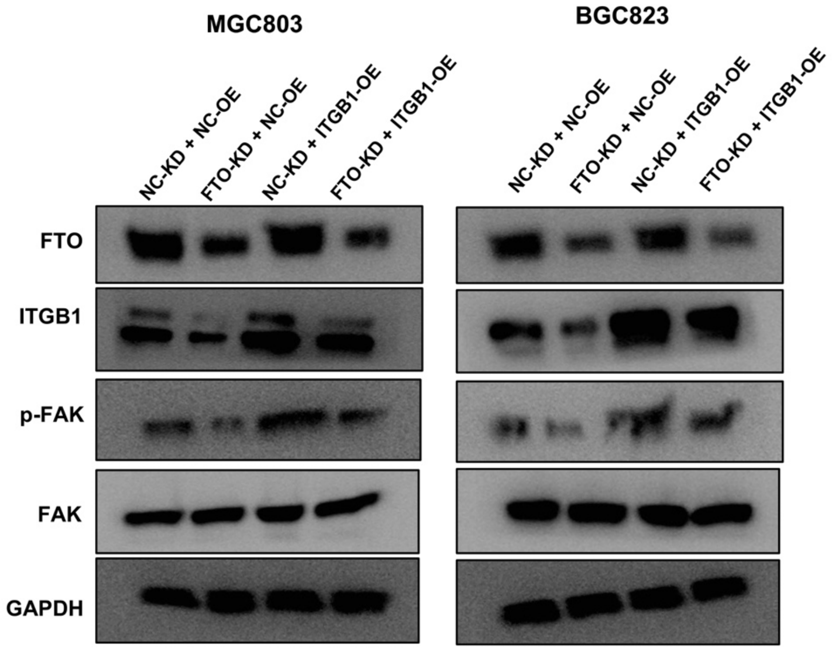

Figure S7 The downstream pathway protein in GC cells after co-transfected with siNC or FTO siRNAs and ITGB1 plasmids or empty vectors were detected by Western blot. GAPDH was used as an internal control for all western blot assays.

Table S1 The univariate and multivariate Cox regression of prognostic factors for OS in GSE62254 dataset

	Univariate Cox				Multivariate Cox			
**Gene**	**Coef**	**HR**	**95% CI**	***P***	**Coef**	**HR**	**95% CI**	***P***
Gender	0.042	1	0.73-1.5	0.82				
Age	0.2	1.2	0.87-1.7	0.25				
T	0.61	1.8	1.5-2.3	<0.001	0.36	1.44	1.13-1.83	0.003
N	0.72	2.1	1.7-2.5	<0.001	0.57	1.77	1.45-2.15	<0.001
M	1.4	4	2.5-6.3	<0.001	0.84	2.31	1.44-3.69	<0.001
FTO	0.54	1.7	1.2-2.4	0.0021	0.36	1.43	1.01-2.03	0.041

Table S2 KEGG pathway enrichment analysis of turquoise module

**Category**	**ID**	**Term**	**p.adjust**	**Count**
KEGG	hsa04360	Axon guidance	1.60E-07	54
KEGG	hsa04510	Focal adhesion	2.23E-07	57
KEGG	hsa04512	ECM-receptor interaction	1.03E-06	32
KEGG	hsa00480	Glutathione metabolism	4.85E-05	22
KEGG	hsa04810	Regulation of actin cytoskeleton	0.0002	51
KEGG	hsa05412	Arrhythmogenic right ventricular cardiomyopathy (ARVC)	0.0002	25
KEGG	hsa05205	Proteoglycans in cancer	0.0006	48
KEGG	hsa04151	PI3K-Akt signaling pathway	0.0008	72
KEGG	hsa04015	Rap1 signaling pathway	0.0009	48
KEGG	hsa01230	Biosynthesis of amino acids	0.0010	23
KEGG	hsa05414	Dilated cardiomyopathy (DCM)	0.0011	27
KEGG	hsa04530	Tight junction	0.0013	40
KEGG	hsa05410	Hypertrophic cardiomyopathy (HCM)	0.0021	25
KEGG	hsa05146	Amoebiasis	0.0026	27
KEGG	hsa04010	MAPK signaling pathway	0.0035	59
KEGG	hsa04520	Adherens junction	0.0069	20
KEGG	hsa00561	Glycerolipid metabolism	0.0069	18
KEGG	hsa04022	cGMP-PKG signaling pathway	0.0069	37
KEGG	hsa01200	Carbon metabolism	0.0090	28
KEGG	hsa00220	Arginine biosynthesis	0.0090	9

Table S3 Correlation of FTO and coexpression genes in FA and ECM signaling pathway

Gene	R	*P*	Gene	R	*P*
COL4A4	0.3778	4.661e-12	ITGB4	-0.1108	0.05014
COL4A5	0.4154	1.738e-14	ITGB6	-0.00458	0.9357
COL9A2	-0.2226	7.105e-05	LAMA3	0.001415	0.9801
ITGA1	0.5322	2.719e-24	LAMA5	0.3346	1.267e-09
ITGA2	-0.06198	0.2743	LAMB2	0.4445	1.372e-16
ITGA3	0.03187	0.5743	LAMB3	-0.2172	0.0001069
ITGA5	0.3946	4.21e-13	LAMC1	0.5006	2.963e-21
ITGA6	-0.1639	0.003631	LAMC2	-0.07985	0.1587
ITGA7	0.5265	1.013e-23	THBS3	0.4346	7.528e-16
ITGA8	0.3236	4.602e-09	THBS4	0.4934	1.318e-20
ITGA9	0.5239	1.857e-23	TNC	0.49	2.61e-20
ITGAV	0.3893	9.024e-13	TNXB	0.4783	2.661e-19
ITGB1	0.508	6.126e-22	VTN	0.05902	0.2979
